# Mandibular Fractures Admitted to the Emergency Department: Data Analysis from a Swiss Level One Trauma Centre

**DOI:** 10.1155/2016/3502902

**Published:** 2016-08-30

**Authors:** Kemal Yildirgan, Edris Zahir, Siamak Sharafi, Sufian Ahmad, Benoit Schaller, Meret E. Ricklin, Aristomenis K. Exadaktylos

**Affiliations:** ^1^Department of Emergency Medicine, Bern University Hospital, Freiburgstrasse, Bern, Switzerland; ^2^Thoracic Surgery, Department for Stomach-Intestines, Liver and Lung Diseases, Bern University Hospital, Freiburgstrasse, Bern, Switzerland; ^3^Department of Cranio-Maxillofacial Surgery, Bern University Hospital, Bern, Switzerland

## Abstract

Mandibular fracture is a common occurrence in emergency medicine and belongs to the most frequent facial fractures. Historically road traffic injuries (RTIs) have played a prominent role as a cause for mandibular fractures. We extracted data from all patients between August 2012 and February 2015 with “lower jaw fracture” or “mandibular fracture” from the routine database from the emergency department. We conducted a descriptive analysis at a Swiss level one trauma centre. 144 patients were admitted with suspected mandibular fractures. The majority underwent CT diagnostic (83%). In 7% suspected mandibular fracture was not confirmed. More than half of all patients suffered two or more fractures. The fractures were median or paramedian in 77/144 patients (53%) and in other parts (corpus, mandibular angle, ramus mandibularis, collum, and temporomandibular joint) in 100/144 (69%). Male to female ratio was 3 : 1 up to 59 years of age; 69% were younger than 40 years. 72% of all patients presented during daytime, 69% had to be hospitalized, and 31% could be discharged from the ED after treatment. Most fractures were due to fall (44%), followed by interpersonal violence (25%) and sport activities (12%). Falls were a dominant cause of fracture in all age groups while violence and sport activities were common only in younger patients. Comparisons to other studies were difficult due to lack of standardization of causes contributing to the injuries. In the observed time period and setting RTIs have played a minor role compared to falls, interpersonal violence, and sports. In the future, standardized documentation as well as categorization of causes for analytic purposes is urgently needed to facilitate international comparison of studies.

## 1. Introduction

The bones of the face are the most exposed part of the body and are therefore particularly vulnerable in road traffic injuries (RTIs) or deliberate violence [1]. In lower-income and newly industrialized countries such as Jordan, Nigeria, Brazil, India, and Egypt, RTIs are the most frequent cause of mandibular fractures [2–6], while in the USA and Canada, Australia and New Zealand, and European countries, interpersonal violence is most frequently responsible [7–12].

The mandible is one of the most frequently fractured facial bones and is involved in 36–70% of all facial fractures [13–15]. This is a much higher incidence than other facial bones and is due to its general mobility and limited bone support [16]. The characteristics of facial fractures depend on environmental factors, gender, age, and the mechanism of injury, such as an assault, fall, or RTI [17, 18]. The presence of teeth in the mandible is a significant anatomic factor and means that such fractures require a different approach from those elsewhere in the skeleton.

It has been shown that a systematic review of patient records can improve diagnosis and treatment and help to increase the use of preventive interventions such as airbags, seat belts, and a combination of the two [19]. Also, known injury patterns contribute to accurate and prompt diagnosis and treatment in the ED.

The aim of this study was to characterize patients presenting between August 2012 and February 2015 to our level one trauma centre in central Switzerland with suspected or diagnosed lower jaw fracture by documenting age, gender, cause, and anatomic distribution of mandibular fractures.

## 2. Materials and Methods

Using the keywords “lower jaw fracture” and “mandibular fracture,” we queried our routine database (E-care bvba, Turnhout, Belgium) for patients admitted to our emergency department with suspected mandibular fractures between August 2012 and February 2015. We included patients of all ages continuously.  Table 1 shows the characteristics of each case documented.

Bicycle accidents were defined as falls rather than RTAs and the capitulum temporomandibular joint and mandibular condyle were classed as the temporomandibular joint. The number of fractures in and out of alignment were summed to calculate the total.

For case analysis per month only data were used of fully available years (2013 and 2014). However for analysis of time of the day admission all data were analysed. Data were entered in Microsoft Excel (MS Office 2010, Redmond, WA) and to calculate frequencies, percentages, and 2-by-*n* tables the pivot table function of Excel was used.

## 3. Results

Between August 2012 and February 2015 144 patients were admitted for suspected mandibular fracture. The mean age was 38.6 years (range 18–88). Ninety-four (65%) patients were younger than 40 years. Most were Swiss (84%) and male (72%). Demographic details are given in Table 2.

**Figure 1:**
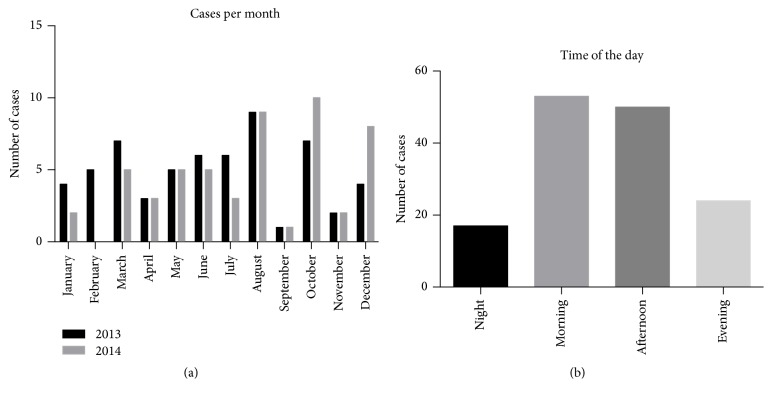
(a) Number of patients with suspected mandibular fracture. The monthly incidence was summed for years 2013 and 2014 (*n* = 112). (b) Number of mandibular fractures by time of day. Morning, 06:00–11:59; afternoon, 12:00–17:59; evening, 18:00–23:59; night, 0:00–05:59.

Seasonal trends in incidence were not apparent, but there were marked differences between individual months when the figures for 2013 and 2104 were totalled: most patients were admitted in August (23/112; 21%), October (19/112; 17%), and December (18/112; 16%), and the fewest in April (6/112; 5%) and September (7/112; 6%) (Figure 1(a)). Most presented during the day (06:00–17:59: 103/144; 72%), with fewer in the evening (18:00–23:59: 24/144; 17%) and at night (00:00–05:59: 17/144; 12%) (Figure 1(b)).

**Figure 2:**
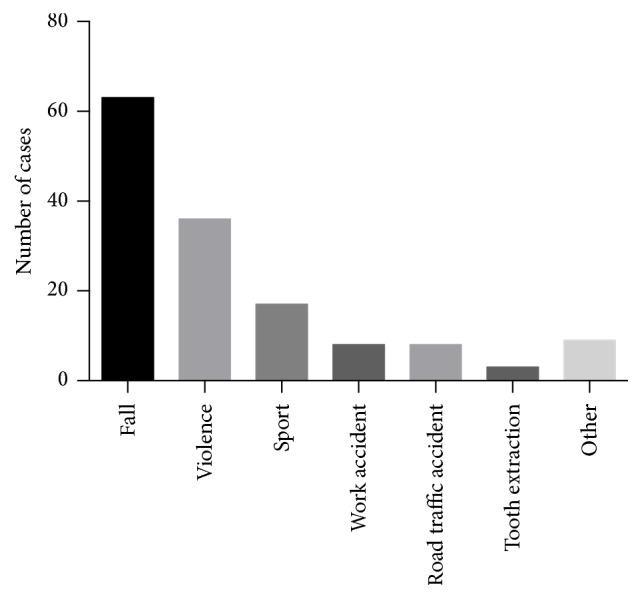
Numbers of patients by cause of fracture.

One hundred (100/144; 69%) patients had to be hospitalized and 44 (31%) were discharged from the ED after treatment. The most frequent causes of mandibular fracture were falls (63/144; 44%), followed by interpersonal violence (36/144; 25%) and sport accidents (17/144; 12%) (Figure 2). Classification of the 16 bicycle accidents as RTI would have lifted the overall proportion of RTI from 6% to 19% and would become the third most frequent cause (behind falls and violence).

After initial clinical examination, the presumed exact sites of the fractures were not recorded; however, the suspected number of fractures could be analysed and compared with the number of fractures after imaging. No fracture (*N* = 10) or a single fracture (*N* = 48) was presumed in 58/144 (40%) patients and two or more fractures were suspected in 86/144 (60%) patients (Table 3). After imaging 22 patients had no fracture, 46 had one, 56 had two, and only 20 patients had 3 or more fractures (Table 3). The frequency distribution of the number of fractures between suspect fractures and documented fractures was not statistically significant (*p* value for heterogeneity = 0.10).

Upon clinical examination 101/144 (70%) patients had an accurate clinical suspicion of the number of fractures (Table 4). The accuracy of the diagnosis did not differ by cause of fracture (*p* value for heterogeneity = 0.83).

Imaging information was not available for 14 patients. 72/130 patients (56%) underwent orthopantogram, 108 (83%) computer tomography, and 46 (35%) both. In 11 cases the clinically suspected fracture was excluded by imaging techniques.

The fractures were median or paramedian in 77/144 patients (53%) and in other parts (corpus, mandibular angle, ramus mandibularis, collum, and temporomandibular joint) in 100/144 (69%), and 33/144 (23%) patients had both types of fracture. Of these, most were located in the collum (42/100 patients, 42%) and the angle (30/100 patients, 30%), with corpus, mandibular joint, and ramus fractures in 15, 14, and 11/100 patients, respectively. Only one pathological fracture and five compound fractures (disrupted skin or mucosa) were observed.

**Figure 3:**
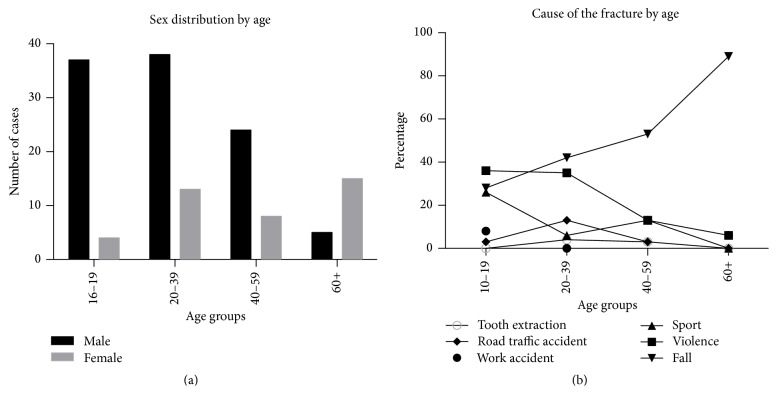
(a) Proportions of men (black) and women (grey) in each age group. (b) Causes of the fracture by age group.

The fracture pattern differed according to age and sex. The ratio of men to women was 3 : 1 up to the age of 59, but then it changed to 1 : 2 in patients older than 60 years (Figure 3(a)).

Falls were the main cause of mandibular fractures in all age groups, especially in patients older than 40 years with the exception for patients below 20 years of age, where violence was slightly more common. Sports activities were also much more frequent in the 16–19 years' age group than in the other age groups.

**Figure 4:**
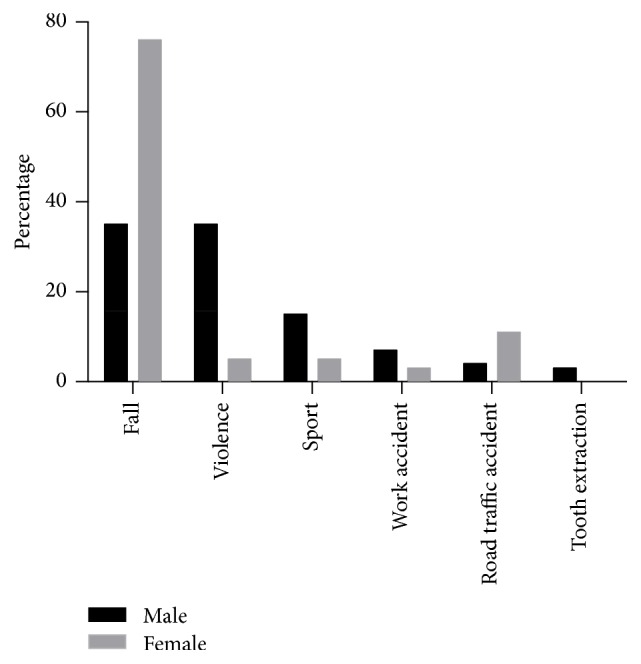
Cause of fracture for men (black) and women (grey) among all patients with one of the specified causes (men *n* = 97; women *n* = 38).

Not only the age but also sex has a large influence of the cause of the accidents. Among all patients, independent of their sex, causes are specified in Table 4 (*N* = 135). In women the leading cause of a suspected fracture was a fall with 76% (29/38). In men 35% (34/97) were due to a fall or violence, followed by sports with 15% (14/97); see Figure 4. In the age group 60+ sixteen patients presented because of a fall, 14 women and 2 men. Eight women and both men were less than 75 years old; six women were at least 75 years old.

**Figure 5:**
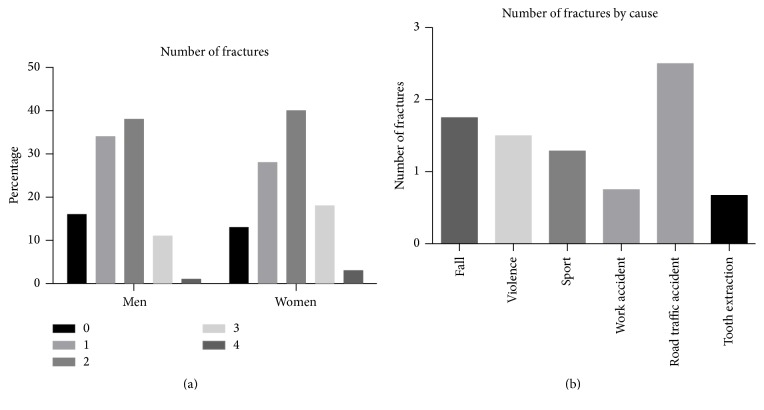
(a) Number of fractures by sex. (b) Average number of fractures by cause.

Each patient suffered an average of 1.6 fractures.  Figure 5(a) shows that sex had no relevant influence on the number of fractures. The cause of the fracture did, however, influence the number of fractures. RTAs caused the highest number of fractures with an average of 2.5 per patient, and tooth extractions the lowest with 0.67 fractures per patient.

**Figure 6:**
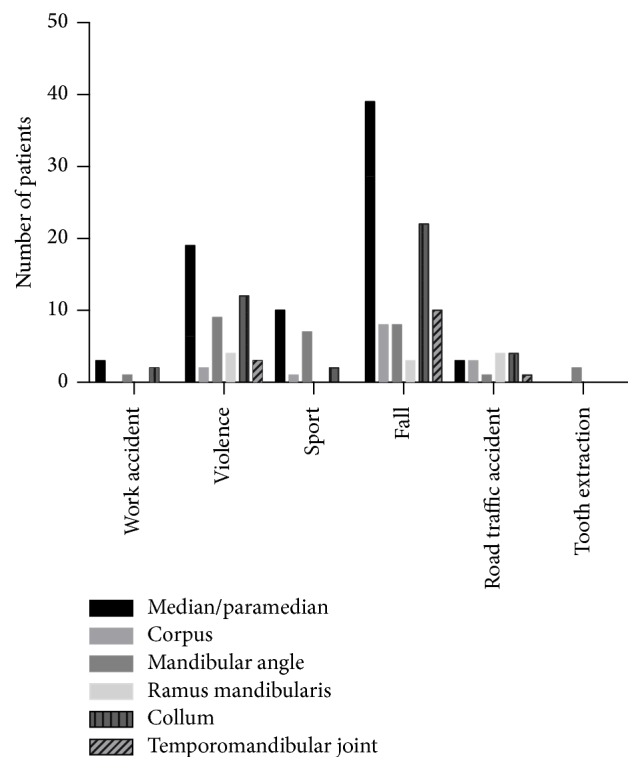
Frequency of fracture sites by cause.

The analysis of the frequency of fracture sites by cause showed that mandibular injuries due to violence mainly led to median/paramedian fractures as well as fractures in the mandibular angle and the collum, sport injuries to median/paramedian fractures and fractures in the mandibular angle, and falls to median/paramedian fractures as well as fractures of the collum (Figure 6).

**Figure 7:**
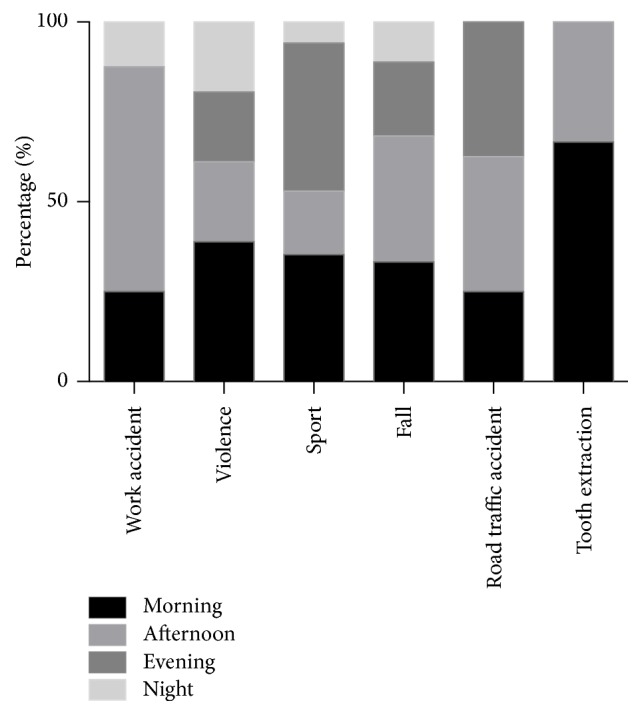
Number of patients presented with suspected mandibular fractures by cause and time of day.

Working accidents happened mainly (88%; 7/8) in the afternoon or at night. The time period where patients with mandibular fractures due to violence presented most frequently was the morning, with similar smaller numbers of patients at other times of day. Fractures due to falls were most frequent during the daytime and were almost half as frequent in the evening and half as frequent again during the night. Fractures due to RTAs mostly occurred in the afternoon and evening, and tooth extraction during daytime only (Figure 7).

## 4. Discussion

The aim of this study was to characterize patients presenting between June 2012 and February 2015 to our emergency department with suspected or diagnosed mandibular fracture. The typical patient with a suspected mandibular fracture is male and younger than 40 years of age [15, 16, 20]. The mean age of our study population is well comparable with that of a multicentre European study of patients with maxillofacial traumas where the mean age among countries ranged between 29.9 and 43.9 years [21] The male : female ratio in our study population was 3 : 1 in patients younger than 60. A high ratio of men to women of 4.4 : 1 and 2.5 : 1 and a high incidence of mandibular fractures in patients aged between 20 and 29 years have been reported elsewhere [15, 22].

We saw no pattern of seasonal fluctuation for the cause of fractures or number of patients treated. Admissions to the ED were most frequent in August and October and least frequent in September and November. A decrease in number of cases has been reported for the fourth trimester, but seasonal variations have not been reported [18]. With regard to the time of presentation, 34% of our patients were admitted in the morning with lower, similar numbers in the afternoon and evening and a distinct drop after midnight. Falls were responsible for just less than half of all fractures in our study, with a quarter due to interpersonal violence and just over 10% as a result of sports trauma. However, the dominant cause in men in our study aged between 16 and 39 was interpersonal violence, responsible for fractures in 40% of men.

In a recent study from Switzerland, RTIs were reported as the most frequent cause of mandibular fractures, followed by sports accidents [22]. In another study from the US mandibular fractures among men stemmed mostly from assault (49.1%) and motor vehicle accidents (25.4%) [23] In contrast, in our data the most frequent underlying cause was falls. Differences may in part result from the fact that the mentioned studies were conducted among hospitalized patients whereas our data were collected from a general accident and emergency department.

Sanger et al. commented that comparisons are difficult because no internationally validated categories have been formulated for the causes of mandibular fractures [24]. The classification of the type of interpersonal violence is problematic because it may involve weapons, and imprecise terminology is used, such as brawls, fights, and assaults synonymously being used in parallel. While interpersonal violence is often reported as the principal cause of mandibular fractures in developed countries [7, 10], RTIs have been reported as the main cause in developing countries [5, 6].

Regarding causes for mandibular injuries and age there are three equally frequent causes in the youngest age group (10–19 years old): sport, violence, and falls (Figure 3(b)). In the next two decades of life “sport” drops out as important cause, and in those older than at least 40 years only “falls” remain as predominant cause. Amongst our patients, falls were a major cause of mandibular fractures in all age groups in both men and women (Figure 3(b)). They are most obviously the predominant cause among the elderly, once because increasing frailty, decreasing physical fitness, and lack of adaptive positional reactivity predispose to uncontrolled falls leading to even mandibular injuries and secondly because other potential causes, such as sports, work accidents, and crude violence are fading in importance. Approximately half of all mandibular injuries due to falls among women occur in those at least 60 years old. Of 14 elderly women (in the age group 60+) who were injured because of a fall six (43%) were at least 75 years old, whereas the two men in that age group who presented in the emergency department because of a fall were “only” 61 and 74 years old. These data are mirroring the increasing gender difference in the older age groups.

Intoxication, domestic violence, and falls from bikes, for example, may also be classified as falls and may therefore also artificially inflate this number, as may interpersonal violence and especially domestic violence reported as a fall.

Only 6% of our patients had mandibular fractures due to RTIs. We have no explanation for this low incidence, especially since other authors have reported much higher incidences, although this does appear to be related to the developmental state of the country [22]. Even in Germany, however, several studies in the past few years have reported that RTIs are responsible for 23 to 32% of mandibular fractures [25, 26]. Similar figures of 22% and 25% have also been reported from Australia and the USA [20, 23].

Among the more frequent causes (violence, sport, and falls) it was apparent that these seemed to predispose to particular fracture sites (as shown in Figure 6). The knowledge of these may help in reaching the correct diagnosis when a patient reports the history of the injury.

Our patients had an average of 1.6 fractures each. Another study has reported similar figures [24]. RTIs were associated with the highest average number of fractures. Such fractures are often complicated, and all of our patients with such fractures had to be hospitalized, indicating the severity of the injury.

The study has the following limitations. (1) It includes only a limited number of patients; in addition data were collected only in a single university hospital; thus we are unable to generalize our findings. (2) As there is no known catchment population we could not calculate incidences. (3) As it is a retrospective study original data were not documented in a standardized fashion and were frequently incomplete. Moreover, there is no agreement upon international categorization of causes, which limits comparability to other studies.

In summary, analysis of data collected during a period of roughly two and half years in a wealthy Swiss city revealed a young age distribution and a predominance of men. RTIs have decreased in importance. The somewhat surprising finding that falls were the most frequent cause for suspected mandibular fractures among both women (by far) and men (together with violence) cannot entirely be explained. In the future, standardized documentation as well as categorization of causes for analytic purposes is urgently needed to facilitate international comparison of studies.

## Figures and Tables

**Table 1:** Characteristics of patients admitted to the emergency department with mandibular fracture.

Characteristic	Details or derived variable
Demographic data	Age 16 and older
	Sex
	Nationality
Day and time of admission	Duration of hospitalization
Day and time of discharge	
Inpatient or outpatient	n.a.
Cause of fracture	Work accident
	Interpersonal violence
	Sport
	Fall
	Road traffic injuries
	Tooth extraction
	Other
Number of suspected fractures	n.a.
Final site of fracture (according to imaging)	Median, paramedian, corpus, mandibular angle, ramus mandibularis, collum, temporomandibular joint
Type of fracture	Compound or closed
Pathological fracture	Yes or no
Side of fracture	
Imaging	Orthopantogram (OPTG)
	Computer tomography (CT)
Confirmation of fracture	Yes or no

n.a. = not applicable.

**Table 2:** Demographic details (*N* = 144).

Variable	*N* (%)
Age (years)	
16–19	41 (28)
20–39	53 (37)
40–59	30 (21)
≥60	20 (14)
Sex	
Male	104 (72)
Female	40 (28)
Nationality	
Swiss	121 (84)
Other	23 (16)

**Table 3:** Number (%) of patients with suspected and documented fractures (*N* = 144).

Number of fractures	Suspected fractures	Documented fractures	Difference
	*N* (%)	*N* (%)	*N*
0	10 (7)	22 (15)	+12
1	48 (33)	46 (32)	−2
2	55 (38)	56 (39)	+1
3	30 (21)	18 (13)	−12
4	1 (1)	2 (1)	+1

**Table 4:** Underestimates and overestimates of number of fractures per patient by cause (*N* = 144).

Cause	*N*	Number of fractures			Discrepancy
		Underestimated	Accurate	Overestimated	*N* (%)^a^
Work accident	8	3	5	0	3 (38)
Road traffic accident	8	2	5	1	3 (38)
Fall	63	10	42	11	21 (33)
Tooth extraction	3	1	2	0	1 (33)
Sport	17	5	12	1	6 (35)
Interpersonal violence	36	7	28	0	7 (19)
Other	9	2	7	0	2 (22)

^a^% of *N* for cause of fracture.
